# Characterising polypharmacy in the very old: Findings from the Newcastle 85+ Study

**DOI:** 10.1371/journal.pone.0245648

**Published:** 2021-01-19

**Authors:** Laurie E. Davies, Andrew Kingston, Adam Todd, Barbara Hanratty

**Affiliations:** 1 Population Health Sciences Institute, Newcastle University, Newcastle upon Tyne, United Kingdom; 2 School of Pharmacy, Newcastle University, Newcastle upon Tyne, United Kingdom; Universidade de Sao Paulo, BRAZIL

## Abstract

**Background:**

Polypharmacy is potentially harmful and under-researched amongst the fastest growing subpopulation, the very old (aged ≥85). We aimed to characterise polypharmacy using data from the Newcastle 85+ Study—a prospective cohort of people born in 1921 who turned 85 in 2006 (n = 845).

**Methods:**

The prevalence of polypharmacy at baseline (mean age 85.5) was examined using cut-points of 0, 1, 2–4, 5–9 and ≥10 medicines—so-called ‘no polypharmacy’, ‘monotherapy’, ‘minor polypharmacy’, ‘polypharmacy’ and ‘hyperpolypharmacy.’ Cross-tabulations and upset plots identified the most frequently prescribed medicines and medication combinations within these categories. Mixed-effects models assessed whether gender and socioeconomic position were associated with prescribing changes over time (mean age 85.5–90.5). Participant characteristics were examined through descriptive statistics.

**Results:**

Complex multimorbidity (44.4%, 344/775) was widespread but hyperpolypharmacy was not (16.0%, 135/845). The median medication count was six (interquartile range 4–8). Preventative medicines were common to all polypharmacy categories, and prescribing regimens were diverse. Nitrates and oral anticoagulants were more frequently prescribed for men, whereas bisphosphonates, non-opioid analgesics and antidepressants were more common in women. Cardiovascular medicines, including loop diuretics, tended to be more frequently prescribed for socioeconomically disadvantaged people (<25^th^ centile Index of Multiple Deprivation (IMD)), despite no difference in the prevalence of cardiovascular disease (p = 0.56) and diabetes (p = 0.92) by IMD.

**Conclusion:**

Considering their complex medical conditions, prescribing is relatively conservative amongst 85-year-olds living in North East England. Prescribing shows significant gender and selected socioeconomic differences. More support for managing preventative medicines, of uncertain benefit, might be helpful in this population.

## Introduction

Polypharmacy is potentially harmful and under-researched amongst the fastest growing section of society, the very old (aged ≥85) [1]. Few studies have explored polypharmacy in this population [2–10], despite medication sensitivity often increasing with the late-life problems of physiological decline, multimorbidity and frailty. Even in the younger old, polypharmacy is most often measured through reductionist categorical definitions that offer no indication of the complexities or appropriateness of prescribing [11].

The contribution of gender to polypharmacy is also seldom studied in the very old [4, 6–9, 12–15]. Women live longer than men with more total disease and disability, along with more chronic versus life-threatening conditions, so prescribing might vary accordingly with gender [16–19]. Gender differences in symptom perception, health seeking behaviour, symptom reporting and therapeutic responses and adverse effects might also influence prescribing [20–22]. We also do not know whether late-life social disadvantage influences prescribing in those aged 85 and over, but it may do so given that socioeconomic status influences health [23–27], and health inequalities amplify across the life course [28, 29].

This study thus characterised polypharmacy in the very old using data from the Newcastle 85+ Study—examining its prevalence, most common medications and medication combinations, as well as prescribing differences across gender and socioeconomic status.

## Methods

### Recruitment and study protocol

Health and medication data were extracted from the Newcastle 85+ Study: a population-based longitudinal study of very old adults living in North East England who were born in 1921, aged 85 in 2006 and permanently registered with one of 53 participating general practices in Newcastle or North Tyneside [17, 30, 31]. When the study began (2006), the sociodemographic composition of the cohort was representative of England and Wales, but participants with end-stage terminal disease were excluded (n = 11) [17]. Data were collected in two ways: multidimensional health assessments and general practice medical records [17, 30–32]. Full details of the questions asked are available at: http://research.ncl.ac.uk/85plus/.

### Ethics approval

The Newcastle 85+ Study was approved by the Newcastle and North Tyneside Local Research Committee One (Ref: 06/Q0905/2), and informed written consent was obtained from all participants.

### Polypharmacy status

The prevalence of polypharmacy at baseline (mean age 85.5, 2006–7) was examined using cut-points of 0, 1, 2–4, 5–9 and ≥10 medicines—so-called ‘no polypharmacy’, ‘monotherapy’, ‘minor polypharmacy’, ‘polypharmacy’ and ‘hyperpolypharmacy’ [33–35]. Cross-tabulations and intersecting set plots [36] then identified the most frequently prescribed medicines and medication combinations within these categories, at baseline. Over-the-counter medications and prescribed items such as vaccines, wound management products and catheter/stoma products were excluded from the above definitions (S1 Table) [37]. All medications were coded using the British National Formulary (58th edition) [38].

### Disease and disability status

Data on fifteen disease groups were analysed to provide a complete picture of multimorbidity in the baseline cohort. Some were analysed individually (e.g. hypertension) and others as composite variables (e.g. a record of any arthritic disease was taken as a diagnosis of arthritis) [16]. Full details of disease status construction and composition can be found in S2 Table. A disability score (0/1-6/7-12/13-17) was assigned from the total number of activities of daily living, instrumental activities of daily living and mobility items performed with any self-reported difficulty. The higher score, the greater the level of disability [39, 40].

### Statistical methods

Socioeconomic position was measured at the small area level via the Index of Multiple Deprivation (IMD)–a weighted construct of income, employment, education, health, crime and the living environment. IMD was cut at the <25th, 25th-75th and >75th centiles in line with previous research [41, 42]. Univariate ordinal logistic regression assessed whether polypharmacy differed by gender and socioeconomic position at baseline. Mixed-effects models then assessed whether these variables were associated with prescribing changes over time (mean age 85.5–90.5 years, baseline to 60 months post-baseline). Only medicines prescribed at a frequency of ≥40 were included, as those with very small sample sizes would have insufficient power for longitudinal analysis, and any gender or socioeconomic differences they may have would not be clinically relevant. Basic health and sociodemographic characteristics were analysed separately by sex using the chi-squared test for descriptive purposes. Gender and socioeconomic differences in disease prevalence were also examined to inform our understanding for prescribing (S3 and S4 Tables). All analyses were performed using R 3.5.0.

## Results

### Key characteristics of study population

The clinical complexity of the Newcastle 85+ baseline cohort has been reported in detail [17, 37]. Briefly, 845 participants were included in this study. Most were women (62.2%, 526/845), educated for 0–9 years (64.4%, 534/829), lived in standard housing, (76.5%, 646/845) had mild disability (score of 1–6, 50.8%, 425/837) and four or more diseases (44.4%, 344/775). A further 43.3% (364/841) were frail, and 49.9% (400/802) experienced pain in the last month (Table 1). The most common diseases were arthritis (66.6%, 563/845), hypertension (57.3%, 484/845) and eye disease (53.4%, 451/845) (S3 Table).

**Table 1:** Health and sociodemographic characteristics of participants at baseline (n = 845).

	All n (%)	Men n (%)	Women n (%)	p-value
**Education (years)**	829			0.576
0–9	534 (64.41)	195 (62.30)	339 (65.70)	
10–11	189 (22.8)	77 (42.60)	112 (21.71)	
≥12	106 (12.79)	41 (13.10)	65 (12.60)	
**Housing**	845			<0.001
Standard	646 (76.45)	266 (83.39)	380 (72.24)	
Sheltered	112 (13.25)	33 (10.34)	79 (15.02)	
Institution	87 (10.30)	20 (6.27)	67 (12.74)	
**Deprivation (IMD) centile**^a^	845			0.025
<25^th^	213 (25.21)	97 (30.41)	116 (22.05)	
25^th^-75^th^	425 (50.29)	149 (46.71)	276 (52.47)	
>75^th^	207 (24.50)	73 (22.88)	134 (25.48)	
**Pain in last month lasting ≥1 days**	400^b^ (49.88)	139 (44.84)	261 (53.05)	0.028
**Frailty (Rockwood 40-item index)**	841			<0.001
Not frail (<0.25)	477 (56.72)	209 (66.14)	268 (51.05)	
Frail (>0.25)	364 (43.28)	107 (33.86)	257 (48.95)	
**Categorised disability score**	837			<0.001
None	164 (19.59)	88 (27.76)	76 (14.62)	
1–6	425 (50.78)	157 (49.53)	268 (51.54)	
7–12	163 (19.47)	47 (14.83)	116 (22.31)	
13–17	85 (10.16)	25 (7.89)	60 (11.54)	
**Disease groups**	775			0.002
0	16 (2.06)	10 (3.29)	6 (1.27)	
1	77 (9.94)	34 (11.18)	43 (9.12)	
2–3	338 (43.61)	149 (49.01)	189 (40.13)	
≥4	344 (44.39)	111 (36.51)	233 (49.47)	

^a^ < 25^th^ most disadvantaged, >75^th^ most affluent

^b^ n = 802

In the month prior to baseline health assessment, polypharmacy (49.6%, 419/845) was more common than minor polypharmacy (24.6%, 208/845) and hyperpolypharmacy (16.0%, 135/845) (Table 2). The median medication count was six (IQR 4–8).

**Table 2:** Prevalence of polypharmacy amongst participants at baseline (n = 845).

Medication count	Definition	All n (%)	Men n (%)	Women n (%)
0	No polypharmacy	39 (4.62)	18 (5.64)	21 (3.99)
1	Monotherapy	44 (5.21)	22 (6.90)	22 (4.18)
2–4	Minor polypharmacy	208 (24.62)	75 (23.51)	133 (25.29)
5–9	Polypharmacy	419 (49.59)	170 (53.29)	249 (47.34)
≥10	Hyperpolypharmacy	135 (15.98)	34 (10.66)	101 (19.20)

### Most commonly prescribed medicines within polypharmacy categories

Table 3 presents the twenty most frequently prescribed medicines amongst people with minor, polypharmacy and hyperpolypharmacy at baseline. Cardiovascular medications (including statins, aspirin and beta-blockers), non-opioid analgesics and proton-pump inhibitors were among the ten most frequently prescribed medicines in all three categories. Subjects of prescribing indicators including anxiolytics, antipsychotics and urinary antispasmodics were sparse. Antidepressant and anti-dementia medications were also uncommon.

**Table 3:** Top 20 BNF-coded medications within each polypharmacy category at baseline (n = 845).

Minor polypharmacy (n = 208)		Polypharmacy (n = 419)		Hyperpolypharmacy (n = 135)	
Medication	% (n)	Medication	% (n)	Medication	% (n)
Non-opioid analgesics	26.44 (55)	Statins	49.88 (209)	Non-opioid analgesics	74.07 (100)
Aspirin	24.52 (51)	Aspirin	49.16 (206)	Statins	64.44 (87)
Thiazide and related diuretics	21.15 (44)	Non-opioid analgesics	40.57 (170)	Aspirin	57.04 (77)
Statins	21.15 (44)	Beta-blockers	34.37 (144)	Loop diuretics	54.81 (74)
Calcium-channel blockers	16.35 (34)	ACE inhibitors ^a^	31.5 (132)	Proton pump inhibitors	43.7 (59)
ACE inhibitors ^a^	15.38 (32)	Calcium-channel blockers	30.07 (126)	Stimulant laxatives	38.52 (52)
Beta-blockers	12.01 (25)	Proton pump inhibitors	24.34 (102)	Beta-blockers	36.3 (49)
Proton pump inhibitors	10.1 (21)	Loop diuretics	23.63 (99)	ACE inhibitors ^a^	34.81 (47)
Vitamin D with calcium	9.62 (20)	Thiazides and related diuretics	20.53 (86)	Calcium-channel blockers	34.81 (47)
Thyroid hormones	6.73 (14)	Nitrates	18.85 (79)	Nitrates	34.07 (46)
Selective beta-2 agonists	5.77 (12)	Vitamin D with calcium	18.38 (77)	Vitamin D with calcium	31.11 (42)
Tear deficiency, ocular lubricants and astringents	5.77 (12)	Bisphosphonates	14.32 (60)	Bisphosphonates	29.63 (40)
Loop diuretics	5.29 (11)	Stimulant laxatives	14.08 (59)	Opioid analgesics	25.93 (35)
Osmotic laxatives	4.33 (9)	Thyroid hormones	11.46 (48)	Emollients	25.93 (35)
Oral anti-coagulants	4.33 (9)	Selective beta-2 agonists	10.98 (46)	Osmotic laxatives	23.7 (32)
Bisphosphonates	4.33 (9)	Angiotensin-11 receptor antagonists	10.26 (43)	Selective beta-2 agonists	22.96 (31)
Emollients	4.33 (9)	Oral anti-coagulants	10.26 (43)	Thyroid hormones	21.48 (29)
Angiotensin-11 receptor antagonists	3.85 (8)	Emollients	10.26 (43)	Tear deficiency, ocular lubricants and astringents	20.74 (28)
Opioid analgesics	3.85 (8)	Cardiac glycosides	9.55 (40)	Skeletal muscle relaxants	19.26 (26)
Skeletal muscle relaxants	3.85 (8)	Osmotic laxatives	8.83 (37)	Cardiac glycosides	17.78 (24)
Vitamin B_12_	3.85 (8)	-	-	-	-

^a^ Angiotensin-converting enzyme (ACE) inhibitors

### Polypharmacy combinations

At baseline, polypharmacy regimens were highly individualised but often included cardiovascular and analgesic medications. Aspirin and statins were most commonly co-prescribed amongst people with minor polypharmacy and polypharmacy. Non-opioid analgesics, statins, aspirin and loop diuretics were most common in hyperpolypharmacy.

### Gender differences in medication prescription

At baseline, and across the full polypharmacy spectrum, women were 37% (95% CI: 5%-77%) more likely to belong to the monotherapy plus all other categories of polypharmacy vs none than men. Longitudinally, gender differences in individual medications were also observed in mixed-effects models adjusted for deprivation. Tear deficiency medications (OR: 10.54, 95% CI: 3.78–29.39), bisphosphonates (OR: 6.83, 95% CI: 3.45–13.52), vitamin D with calcium (OR: 6.11, 95% CI: 3.70–10.09), non-opioid analgesics (OR: 2.54, 95% CI: 1.85–3.50), topical non-steroidal anti-inflammatory drugs (NSAIDs) (OR: 2.02, 95% CI: 1.05–3.88), selective serotonin reuptake inhibitors (SSRIs) (OR: 2.86, 95% CI: 1.38–5.94), thiazides and related diuretics (OR: 2.54, 95% CI: 1.29–4.98), tricyclic and related antidepressants (OR: 2.34, 95% CI: 1.01–5.44) and emollients (OR: 1.78, 95% CI: 1.12–2.82) showed a significant difference in favour of women. Opioid analgesics, osmotic and stimulant laxatives were also more common in women, but not significantly so. Meanwhile, oral anticoagulants (OR: 0.36, 95% CI: 0.17–0.79) and nitrates (OR: 0.32, 95% CI: 0.15–0.65) were more frequently prescribed in men (S5 Table).

### Socioeconomic differences in medication prescription

At baseline, compared to the referent 25th-75th centile, there was a non-significant difference in polypharmacy between those most affluent (>75th centile IMD) (OR: 1.04, 95% CI: 0.76–1.42) and people living in socioeconomic disadvantage (<25th centile IMD) (OR: 1.03, 95% CI: 0.76–1.39). However longitudinally, mixed-effects models adjusted for sex identified subtle socioeconomic differences in prescribing. People in the most affluent group were prescribed significantly more selective beta-2 agonists (OR: 2.33, 95% CI: 1.02–5.36) and tricyclic and related antidepressants (OR: 2.98, 95% CI: 1.15–7.75) compared to the referent, but less calcium-channel blockers (OR: 0.41, 95% CI: 0.19–0.92) and ACE inhibitors (OR: 0.52, 95% CI: 0.28–0.96). Opioid (OR: 1.13, 95% CI: 0.68–1.87) and non-opioid analgesics (OR: 1.22, 95% CI: 0.85–1.75) were also more common amongst the most affluent, but not significantly so.

The most disadvantaged (<25th centile IMD) were prescribed significantly less topical NSAIDs (OR: 0.37, 95% CI 0.17–0.84) but tended to receive more SSRIs (OR: 1.44, 95% CI: 0.65–3.19) and cardiovascular medicines including statins (OR: 1.38, 95% CI: 0.75–2.52), beta-blockers (OR: 1.23, 95% CI: 0.59–2.58) and loop diuretics (OR: 1.25, 95% CI: 0.74–2.10) (S6 Table), despite no difference in the prevalence of cardiovascular disease (p = 0.56) and diabetes (p = 0.92) across each IMD centile (S4 Table).

## Discussion

### Summary

Polypharmacy is common and highly individualised in the Newcastle 85+ cohort (n = 845), driven by both long-term preventative and symptomatic medicines, as well as significant gender and subtle socioeconomic differences. That said, considering the clinical complexity of the sample in terms of widespread multimorbidity and geriatric syndromes, prescribing for this population was relatively conservative.

### Comparison with existing literature

Prescribing is said to increase with age [43, 44] and polypharmacy was indeed common in the Newcastle 85+ cohort. Almost half of participants took five to nine medicines (49.6%, 419) with a median of six—a slightly higher number than that of other comparable old age cohorts [45, 46]. This may reflect international differences in multimorbidity and prescribing patterns [37, 47]. ‘Six’ may seem a high number of medicines to take but given the many drivers for polypharmacy in our sample, including complex multimorbidity, chronic pain, disease-specific clinical guidelines and health service use [37, 48–51], we could argue that prescribing was relatively contained. With deprescribing in its infancy at study inception (2006) [52], reasons for this conservatism might include survivorship, historic precedent [53] or efforts to avoid adverse drug reactions [54], given their greater likelihood in later life [55, 56] where frailty, renal impairment and cognitive impairment are common.

The contribution of preventative and symptomatic treatments to polypharmacy is consistent with previous research [5, 57] and the challenges of prescribing to people in advanced age. ‘Preventative’ statins could, for example, partly reflect the difficulty in recognising when the end-of-life is approaching, limited time-to-benefit information or the aforementioned dearth of evidence for deprescribing [58, 59], and ‘symptomatic’ non-opioid analgesics and laxatives, the prevalence of pain and reduced mobility in later life [40, 48]. Some of the most frequently prescribed medicines are also ‘high risk.’ ACE inhibitors, beta-blockers and loop diuretics have all been linked to unplanned hospital admissions in younger populations, for example [60]. That said, encouraging prescribing trends were also observed. Subjects of prescribing indicators including anxiolytics, urinary antispasmodics and antipsychotics were infrequently prescribed.

Many of the observed gender differences in prescribing can be explained by differences in disease prevalence and perception. Polypharmacy was more common amongst very old women as a likely consequence of their greater morbidity and poorer self-rated health [17]. Women also used more bisphosphonates (OR: 6.83, 95% CI: 3.45–13.52), non-opioid analgesics (OR: 2.54, 95% CI: 1.85–3.50) and SSRIs (OR: 2.86, 95% CI: 1.38–5.94) for osteoporosis, arthritic diseases and depression, whereas men likely used more nitrates (OR: 0.32, 95% CI: 0.15–0.65) and oral anticoagulants (OR: 0.36, 95% CI: 0.17–0.79) for ischaemic heart disease and atrial fibrillation within the composite cardiovascular disease variable (S3 Table) [17].

Reasons for the socioeconomic differences in prescribing are less clear (S6 Table). The greater prescription of tricyclic (and related) antidepressants (OR: 2.98, 95% CI: 1.15–7.75) to those more affluent (>75th centile IMD) may reflect inequitable pain management. Topical NSAIDs (OR: 0.37, 95% CI 0.17–0.84) were also less common amongst disadvantaged people (<25^th^ centile IMD). Reasons for this are unproven but could include socioeconomic advantage lessening prescribers’ perception of risk, or differences in symptom reporting and access to specialist care. Greater prescription of selective beta-2 agonists to the >75^th^ group might similarly suggest that their overuse is monitored more closely amongst those living in socioeconomic disadvantage, who may be perceived to be at greater risk of associated exacerbation or death [61]. The tendency for disadvantaged people (<25th centile IMD) to use more SSRIs (OR: 1.44, 95% CI: 0.65–3.19) may reflect poorer social support, coping mechanisms and greater stress [62], or an increased vulnerability to prolonged or recurrent late-life depression [63]. Though no difference in the prevalence of cardiovascular disease (p = 0.56) and diabetes (p = 0.92) across each IMD centile, socioeconomically disadvantaged people also tended to be prescribed more of the cardiovascular medicines including ACE inhibitors and calcium-channel blockers, statins, beta-blockers and oral anticoagulants, whose risks can outweigh the benefits in later life [60, 64–66]. Thus, limited deprescribing in the very old may particularly impact deprived individuals.

Finally, polypharmacy combinations were diverse, reflecting the heterogeneity of multimorbidity [37] and propensity for other determinants of medication use,–such as renal function, frailty and medication tolerability -, to vary from person to person in very late life. Indeed, ‘one size does not fit all’ when it comes to polypharmacy [67].

### Strengths and limitations

Our study has several strengths. We characterised polypharmacy in a cohort of 85+ year olds and examined gender and socioeconomic differences in late-life prescribing, thus adding to the scarce literature in these areas. Our pragmatic definition for polypharmacy helped to avoid information loss, and visualising medication combinations helped to communicate prescribing complexity. Finally, we analysed medication and disease data from general practice records as opposed to the less reliable method of self-report [17].

We were unable to judge whether prescribing was potentially inappropriate as we had no information on dosages, durations or current indications; lacked many drug names by chemical substance and aggregate (rather than individual level data) was used. Some findings might also reflect former prescribing practices as the study started in 2006. For example, aspirin was prescribed more often than clopidogrel [68, 69]. The extent to which non-adherence influenced prescribing is also unclear. We could not measure prescribing outside of data collection points, or assess disease severity, so the mixed-effects models may not reflect the true extent to which gender and socioeconomic position influence late-life prescribing [70]. There may have also been a survivor effect longitudinally, but adjustment for mortality made no difference in a sensitivity analysis. As is customary in prescribing research, over-the-counter medicines were excluded from our polypharmacy definition to make our results directly applicable to primary care practice, but we acknowledge that this can lead to an under-estimation of medication use.

### Implications for practice and conclusion

Considering their complex clinical conditions, prescribing is relatively conservative amongst 85-year olds living in North East England. That said, more support for managing preventative medicines, of uncertain benefit and harm, might be helpful in this population. Pharmacists in primary care teams may be an ideal way to provide this. In highlighting prescribing heterogeneity, our findings also underscore the need for personalised healthcare in the very old. This could help to address the significant differences in prescribing prevalence for gender, and the subtle but potentially important differences for socioeconomic position.

## Supporting information

S1 Table Prescribed items excluded from polypharmacy definition. (DOCX)

S2 Table Definitions of disease groups in the Newcastle 85+ baseline cohort [16]. (DOCX)

S3 Table Gender differences in disease prevalence at baseline. (DOCX)

S4 Table Socioeconomic differences in disease prevalence at baseline. (DOCX)

S5 Table Gender differences in prescribing with mixed effects models adjusted for deprivation. (DOCX)

S6 Table Socioeconomic differences in prescribing with mixed effects models adjusted for sex. (DOCX)
