# Supplementary material for: Characterising polypharmacy in the very old: Findings from the Newcastle 85+ Study
Source: PLoS One. 2021 Jan 19;16(1):e0245648. doi: 10.1371/journal.pone.0245648 (PMC7815158; doi:10.1371/journal.pone.0245648)
Supplement: S1 Table — (DOCX) [file pone.0245648.s001.docx]

**S1 Table: Prescribed items excluded from polypharmacy definition**

| **Prescribed item** | **BNF code** |
| --- | --- |
| Stoma products  Peak flow meters, inhaler devices and nebulisers  Hypodermic equipment (excluding lancets)  Diagnostic and monitoring agents for diabetes (including lancets)  Ring/shelf pessary  Electrolytes and water - water for injections  Wound dressings  Tubular bandages, compression hosiery and applicators  Diphtheria vaccine  Influenza vaccine  Typhoid vaccine  Pneumococcal vaccine  Urinary catheter/sheath/leg bag  Anal plug for bowel incontinence  Truss - elastic band  Borderline substances - food  Syringe for injection  KY jelly  Sharps bin  Gloves | 10800  30150  60113  60160  70110  90221  131300  131310  140404  140410  140419  140420  180500  180501  180600  180700  180800  180801  180802  180803 |
