# Supplementary material for: Characterising polypharmacy in the very old: Findings from the Newcastle 85+ Study
Source: PLoS One. 2021 Jan 19;16(1):e0245648. doi: 10.1371/journal.pone.0245648 (PMC7815158; doi:10.1371/journal.pone.0245648)
Supplement: S2 Table — (DOCX) [file pone.0245648.s002.docx]

**S2 Table: Definitions of disease groups in the Newcastle 85+ baseline cohort [16]**

| **Disease group** | **Included diseases** |
| --- | --- |
| **Arthritis** | Ankylosing spondylitis; cervical spondylosis; rheumatoid, degenerative, poly, gouty, septic, peri arthritis; generalised osteoarthritis; hand, hip or knee osteoarthritis; lumbar spondylosis or psoriatic arthropathy |
| **Cancer** | Any cancer within 5 years of diagnosis, excluding non-melanoma skin cancer |
| **Cardiovascular disease** | Angina, coronary angioplasty, coronary artery bypass graft/stent, heart failure, myocardial infarction, atrial fibrillation or atrial flutter |
| **Cerebrovascular disease** | Carotid endarterectomy, stroke or transient ischaemic attack |
| **Cognitive impairment** | Standardised Mini-Mental State Examination (sMMSE) score ≤21, Alzheimer’s disease or dementia |
| **Depression** | Presence of depression in the last 12 months |
| **Diabetes** | Type 1 diabetes, type 2 diabetes or unspecified diabetes |
| **Eye disease** | Cataracts, cataract surgery, age-related macular degeneration, glaucoma, diabetic eye disease, registered partially sighted or registered blind |
| **Hypertension** | Any recorded diagnosis of hypertension |
| **Liver disease** | Abnormal liver function tests (without diagnostic label), non-alcoholic fatty liver disease or autoimmune hepatitis |
| **Osteoporosis** | Osteoporosis |
| **Parkinson’s disease** | Parkinson’s disease |
| **Renal impairment** | eGFR<30ml/min/1.73m^2^ |
| **Respiratory disease** | Fibrosing alveolitis, asbestosis, asthma, bronchiectasis, chronic bronchitis, COPD, emphysema, pneumoconiosis or pulmonary fibrosis |
| **Thyroid disease** | Hyperthyroidism or hypothyroidism |
