# Supplementary material for: Characterising polypharmacy in the very old: Findings from the Newcastle 85+ Study
Source: PLoS One. 2021 Jan 19;16(1):e0245648. doi: 10.1371/journal.pone.0245648 (PMC7815158; doi:10.1371/journal.pone.0245648)
Supplement: S3 Table — (DOCX) [file pone.0245648.s003.docx]

**S3 Table: Gender differences in disease prevalence at baseline**

| **Disease** | **Men n (%)** | **Women n (%)** | **All n (%)** | **p-value ^a^** |
| --- | --- | --- | --- | --- |
| Arthritis | 190 (59.56) | 373 (70.91) | 563 (66.63) | 0.001 |
| Hypertension | 168 (52.66) | 316 (60.08) | 484 (57.28) | 0.041 |
| Eye disease | 147 (46.08) | 304 (57.79) | 451 (53.37) | 0.001 |
| Cardiovascular disease | 158 (49.53) | 223 (42.40) | 381 (45.09) | 0.051 |
| Respiratory disease | 71 (22.26) | 120 (22.81) | 191 (22.60) | 0.918 |
| Cerebrovascular disease | 78 (24.45) | 100 (19.01) | 178 (21.07) | 0.073 |
| Thyroid disease | 21 (6.58) | 100 (19.01) | 121 (14.32) | <0.001 |
| Diabetes | 46 (14.42) | 66 (12.55) | 112 (13.25) | 0.501 |
| Osteoporosis | 13 (4.08) | 99 (18.82) | 112 (13.25) | <0.001 |
| Cognitive impairment | 41 (12.85) | 89 (16.92) | 130 (15.38) | 0.136 |
| Depression | 17 (5.33) | 45 (8.56) | 62 (7.34) | 0.108 |
| Cancer | 23 (7.21) | 23 (4.37) | 46 (5.44) | 0.108 |
| Renal impairment ^b^ | 7 (2.30) | 16 (3.40) | 23 (2.97) | 0.509 |
| Parkinson’s disease | 7 (2.19) | 7 (1.33) | 14 (1.66) | 0.499 |
| Liver disease | 6 (1.88) | 6 (1.14) | 12 (1.42) | 0.384 ^c^ |

^a^ Chi-squared test.

^b^ n = 775.

^c^ Fisher’s exact test.
