# Supplementary material for: Characterising polypharmacy in the very old: Findings from the Newcastle 85+ Study
Source: PLoS One. 2021 Jan 19;16(1):e0245648. doi: 10.1371/journal.pone.0245648 (PMC7815158; doi:10.1371/journal.pone.0245648)
Supplement: S4 Table — (DOCX) [file pone.0245648.s004.docx]

**S4 Table: Socioeconomic differences in disease prevalence at baseline**

| **Disease group** | **<25^th^ centile IMD n (%)** | **25^th^-75^th^ centile IMD n (%)** | **>75^th^ centile IMD n (%)** | **All n (%)** | **p-value ^a^** |
| --- | --- | --- | --- | --- | --- |
| Arthritis | 141 (66.20) | 287 (67.53) | 135 (65.22) | 563 (66.63) | 0.836 |
| Hypertension | 126 (59.15) | 252 (59.29) | 106 (51.21) | 484 (57.28) | 0.127 |
| Eye disease | 206 (49.77) | 231 (54.35) | 114 (55.07) | 451 (53.37) | 0.468 |
| Cardiovascular disease | 94 (44.13) | 199 (46.92) | 88 (42.51) | 381 (45.09) | 0.563 |
| Respiratory disease | 41 (19.25) | 94 (22.12) | 56 (27.05) | 191 (22.60) | 0.152 |
| Cerebrovascular disease | 38 (17.84) | 93 (21.88) | 47 (22.71) | 178 (21.07) | 0.399 |
| Thyroid disease | 30 (14.08) | 63 (14.82) | 28 (13.53) | 121 (14.32) | 0.903 |
| Diabetes | 30 (14.08) | 55 (12.94) | 27 (13.04) | 112 (13.25) | 0.918 |
| Osteoporosis | 23 (10.80) | 61 (14.35) | 28 (13.53) | 112 (13.25) | 0.455 |
| Cognitive impairment | 16 (7.51) | 65 (15.29) | 49 (23.67) | 130 (15.38) | <0.001 |
| Depression | 19 (8.92) | 27 (6.35) | 16 (7.73) | 62 (7.34) | 0.487 |
| Cancer | 13 (6.10) | 30 (7.06) | 3 (1.45) | 46 (5.44) | 0.013 |
| Renal impairment ^b^ | 7 (3.41) | 9 (2.33) | 7 (3.82) | 23 (2.97) | 0.559 |
| Parkinson’s disease | 5 (2.35) | 4 (0.94) | 5 (2.42) | 14 (1.66) | 0.194 ^c^ |
| Liver disease | 4 (1.88) | 6 (1.41) | 2 (0.97) | 12 (1.42) | 0.805 ^c^ |

^a^ Chi-squared test.

^b^ n = 775.

^c^ Fisher’s exact test.
