# Supplementary material for: Characterising polypharmacy in the very old: Findings from the Newcastle 85+ Study
Source: PLoS One. 2021 Jan 19;16(1):e0245648. doi: 10.1371/journal.pone.0245648 (PMC7815158; doi:10.1371/journal.pone.0245648)
Supplement: S5 Table — (DOCX) [file pone.0245648.s005.docx]

**S5 Table: Gender differences in prescribing with mixed effects models adjusted for deprivation**

| **Medication** | **OR (95% CI) women to men** |
| --- | --- |
| Tear deficiency, lubricants and astringents | 10.54 (3.78 - 29.39) |
| Bisphosphonates | 6.83 (3.45 - 13.52) |
| Vitamin D with calcium | 6.11 (3.70 - 10.09) |
| SSRIs | 2.86 (1.38 - 5.94) |
| Thiazides and related diuretics | 2.54 (1.29 - 4.98) |
| Non-opioid analgesics | 2.54 (1.85 - 3.50) |
| Tricyclic and related antidepressants | 2.34 (1.01 - 5.44) |
| Topical NSAIDs | 2.02 (1.05 - 3.88) |
| Emollients | 1.78 (1.12 - 2.82) |
| Thyroid hormones | - |
| Inhaled corticosteroids | 1.66 (0.70 - 3.92) |
| Cardiac glycosides | 1.59 (0.69 - 3.69) |
| Osmotic laxatives | 1.56 (0.99 - 2.45) |
| Opioids | 1.49 (0.96 - 2.30) |
| Selective beta-2 agonists | 1.43 (0.69 - 3.00) |
| Beta-blockers | 1.42 (0.75 - 2.69) |
| Oral iron | 1.41 (0.83 - 2.39) |
| Stimulant laxatives | 1.26 (0.86 - 1.86) |
| Hypnotics | 1.12 (0.50 - 2.49) |
| Proton pump inhibitors | 1.07 (0.64 - 1.79) |
| Skeletal muscle relaxants | 1.07 (0.38 - 2.97) |
| Clopidogrel | 1.07 (0.43 - 2.68) |
| Compound alginates | 1.06 (0.40 - 2.78) |
| Angiotensin-11 receptor blockers | 1.74 (0.40 - 7.56) |
| Oral anticoagulants | 0.36 (0.17 - 0.79) |
| Nitrates | 0.32 (0.15 - 0.65) |
| Aspirin | 0.95 (0.62 - 1.47) |
| Calcium-channel blockers | 0.94 (0.50 - 1.77) |
| Loop diuretics | 0.94 (0.60 - 1.47) |
| Statins | 0.93 (0.56 - 1.54) |
| Vitamin B_12_ | 0.82 (0.36 - 1.84) |
| ACE inhibitors | 0.73 (0.44 - 1.20) |
| Topical corticosteroids with antimicrobials | 0.69 (0.41 - 1.15) |
| Alpha blockers for hypertension | 0.58 (0.21 - 1.64) |
