# Supplementary material for: Characterising polypharmacy in the very old: Findings from the Newcastle 85+ Study
Source: PLoS One. 2021 Jan 19;16(1):e0245648. doi: 10.1371/journal.pone.0245648 (PMC7815158; doi:10.1371/journal.pone.0245648)
Supplement: S6 Table — (DOCX) [file pone.0245648.s006.docx]

**S6 Table: Socioeconomic differences in prescribing with mixed effects models adjusted for sex**

| **Medication** | **OR (95% CI) <25^th^ to**  **25^th^-75^th^ centile IMD** | **OR (95% CI) >75^th^ to**  **25^th^-75th centile IMD** |
| --- | --- | --- |
| Tricyclic and related antidepressants | 0.78 (0.29 - 2.13) | 2.98 (1.15 - 7.75) |
| Selective beta-2 agonists | 0.77 (0.31 - 1.89) | 2.33 (1.02 - 5.36) |
| Non-opioid analgesics | 0.82 (0.57 - 1.19) | 1.22 (0.85 - 1.75) |
| Opioids | 0.94 (0.57 - 1.58) | 1.13 (0.68 - 1.87) |
| Topical corticosteroids with antimicrobials | 0.92 (0.49 - 1.71) | 1.01 (0.54 - 1.88) |
| Topical NSAIDs | 0.37 (0.17- 0.84) | 0.57 (0.27 - 1.20) |
| Calcium-channel blockers | 1.23 (0.59 - 2.58) | 0.41 (0.19 - 0.92) |
| ACE inhibitors | 1.04 (0.58 - 1.87) | 0.52 (0.28 - 0.96) |
| SSRIs | 1.44 (0.65 - 3.19) | 0.48 (0.20 - 1.15) |
| Oral anticoagulants | 1.46 (0.60 - 3.55) | 0.43 (0.16 - 1.13) |
| Statins | 1.38 (0.75 - 2.52) | 0.82 (0.44 - 1.50) |
| Beta-blockers | 1.23 (0.59 - 2.58) | 0.48 (0.22 - 1.04) |
| Loop diuretics | 1.25 (0.74 - 2.10) | 0.78 (0.45 - 1.33) |
| Cardiac glycosides | 1.01 (0.38 - 2.67) | 0.65 (0.24 - 1.76) |
| Vitamin B_12_ | 1.03 (0.39 - 2.68) | 0.72 (0.27 - 1.91) |
| Osmotic laxatives | 1.08 (0.64 - 1.81) | 0.97 (0.57 - 1.64) |
| Bisphosphonates | 0.67 (0.33 - 1.37) | 0.70 (0.34 - 1.41) |
| Thiazides and related diuretics | 0.54 (0.25 - 1.17) | 0.50 (0.23 - 1.10) |
| Oral iron | 0.57 (0.30 - 1.08) | 0.82 (0.45 - 1.50) |
| Aspirin | 0.65 (0.39 - 1.10) | 0.74 (0.45 - 1.24) |
| Tear deficiency, lubricants and astringents | 0.97 (0.35 - 2.67) | 0.68 (0.24 - 1.97) |
| Vitamin D with calcium | 1.14 (0.67 - 1.93) | 1.19 (0.71 - 1.99) |
| Emollients | 1.58 (0.94 - 2.66) | 1.45 (0.86 - 2.46) |
| Inhaled corticosteroids | 2.19 (0.80 - 5.98) | 1.88 (0.69 - 5.10) |
| Stimulant laxatives | 1.09 (0.69 - 1.7) | 1.45 (0.93 - 2.27) |
| Hypnotics | 1.11 (0.43 - 2.87) | 1.16 (0.45 - 3.02) |
| Proton pump inhibitors | 1.25 (0.68 - 2.29) | 1.21 (0.66 - 2.23) |
| Nitrates | 1.20 (0.52 - 2.79) | 1.07 (0.45 - 2.55) |
| Alpha blockers for hypertension | 1.62 (0.49 - 5.39) | 0.72 (0.20 - 2.64) |
| Compound alginates | 1.13 (0.36 - 3.49) | 0.98 (0.31 - 3.08) |
| Skeletal muscle relaxants | 1.20 (0.33 - 4.35) | 0.48 (0.15 - 1.53) |
| Thyroid hormones | 1.48 (0.42 - 5.25) | 0.76 (0.19 - 2.99) |
| Angiotensin-11 receptor blockers | 1.35 (0.29 - 6.34) | 0.75 (0.13 - 4.37) |
| Clopidogrel | 0.69 (0.23 - 2.06) | 0.79 (0.27 - 2.34) |
